# Climate Impacts on Extreme Energy Consumption of Different Types of Buildings

**DOI:** 10.1371/journal.pone.0124413

**Published:** 2015-04-29

**Authors:** Mingcai Li, Jun Shi, Jun Guo, Jingfu Cao, Jide Niu, Mingming Xiong

**Affiliations:** 1 Tianjin Climate Center, Tianjin, 300074, China; 2 School of Environmental Science and Engineering, Tianjin University, Tianjin, 300072, China; University of Aveiro, PORTUGAL

## Abstract

Exploring changes of building energy consumption and its relationships with climate can provide basis for energy-saving and carbon emission reduction. Heating and cooling energy consumption of different types of buildings during 1981-2010 in Tianjin city, was simulated by using TRNSYS software. Daily or hourly extreme energy consumption was determined by percentile methods, and the climate impact on extreme energy consumption was analyzed. The results showed that days of extreme heating consumption showed apparent decrease during the recent 30 years for residential and large venue buildings, whereas days of extreme cooling consumption increased in large venue building. No significant variations were found for the days of extreme energy consumption for commercial building, although a decreasing trend in extreme heating energy consumption. Daily extreme energy consumption for large venue building had no relationship with climate parameters, whereas extreme energy consumption for commercial and residential buildings was related to various climate parameters. Further multiple regression analysis suggested heating energy consumption for commercial building was affected by maximum temperature, dry bulb temperature, solar radiation and minimum temperature, which together can explain 71.5 % of the variation of the daily extreme heating energy consumption. The daily extreme cooling energy consumption for commercial building was only related to the wet bulb temperature (R^2^= 0.382). The daily extreme heating energy consumption for residential building was affected by 4 climate parameters, but the dry bulb temperature had the main impact. The impacts of climate on hourly extreme heating energy consumption has a 1-3 hour delay in all three types of buildings, but no delay was found in the impacts of climate on hourly extreme cooling energy consumption for the selected buildings.

## Introduction

Global surface temperature increased 0.4 ~ 0.8°C between the start and the end of the 20^th^ century and the temperature will rise between 1.8 ~ 4.0°C at the end of the 21^st^ century with an increase in extreme events [1]. Climate change is strongly affecting diverse aspects of human society and the natural world. The significant warming climate of the whole world due to the global climate change is considered to have strong effects on a building’s energy requirement or usage as their heating and cooling needs are related to temperature conditions and weather variations [2,3]. Many previous studies have concerned the climate impact on building energy usage in recent years by using cooling/heating degree days [4,5,6], or by simulating energy consumption [7,8,9]. Revealing the impact of climate change on building energy consumption is beneficial for not only making efficient energy saving measures but also reducing pollutant or greenhouse gas emission [3,10,11]. However, the previous studies concerning the climate change impact on energy consumption or demand primarily focused on the average energy usage. To our knowledge, except some previous studies concerning on peak electrical energy demand [12,13], no corresponding study has, so far, been attempted for the extreme energy consumption that is related to the energy saving measures or safety operation of Heating, Ventilation and Air Conditioning (HVAC).

In China, building is playing an extremely important role in the energy demand sector because buildings account for up to 30% of the total national energy consumption (TNEC) and may be projected to contribute 35% till 2020 [8,10,14]. Many effort and measures have been taken to improve the energy efficiency of buildings since 1986 when the first building code was introduced to China [14]. However, the energy efficiency is generally still very low in China. Particularly, heating energy consumption per unit area is 2–4 times higher than developed countries [10]. The lower energy efficiency is to a large extent due to the lack of detailed information on the climate impacts on building energy consumption, efficient energy saving measures, therefore, were not made according to the climate change.

Buildings designed according to climatic condition of past years may become increasingly costly to operate and maintain in the present and future [3]. Under the conditions of changing climate, especially in summer, the buildings will consume more energy but with poorer indoor air quality and lower thermal comfort. Increase in temperature and extreme weather events, temperature swings, changes in relative humidity and solar radiation should be taken into account to ensure that current and future buildings are able to adapt to these changes and thus minimize the potentially destructive impacts, such as energy use and carbon emissions [3]. In the recent 100 years, China is experiencing an apparently increasing temperature, with average surface temperature increasing 0.5~0.8°C that is slightly higher the global average [15]. The increasing rate of temperature change is even more apparent in large cities due to the urban heat island effect [16,17]. The continuous increase of temperature companied with extreme weather or climate events will largely affect the building energy requirement or consumption. However, to our knowledge, responses of building energy usage to climate and its change are very limited in China [8,10,18]. Also, no corresponding study has yet been attempted for the climate impacts on energy consumption for different types of buildings. Previous studies on building energy consumption are mainly concentrated on analyses by using constant increase in the annual average temperature or changes in degree-days [4,6,19]. This may, to some extent, lead to insufficient estimation of energy consumption requirement by degree-days if adjustment of appropriate parameters was not made [2,5] due to the lack of changing information of relevant variables, such as humidity, solar radiation, and wind speed.

In this study, we selected residential, commercial and large venue buildings in a large city, Tianjin, in northern China. As the second largest city in Northern China, Tianjin city has experienced a substantial increase in urban population and construction land in the last 30 years, which has caused apparent urban heat island [16,20]. Due to the heat island effect, the climate change is more intense [16,20], which should be fully considered in the building design and energy use for energy saving and pollutant or greenhouse gas emission reducing. The energy consumption of three buildings was simulated with a simulation tool (Transient System Simulation Program, TRYSNS). Daily or hourly extreme energy consumption was determined by percentile methods and the climate impact on extreme energy consumption was analyzed. This study aimed: (1) to examine the variations of days of extreme energy consumption under the changing climate conditions, (2) to investigate the dominant climatic parameters affecting daily extreme energy consumption for different building types, (3) to study whether there are apparent delays in the effects of climatic parameters on hourly extreme energy consumption.

## Methodology

### Study area

**Fig 1:**
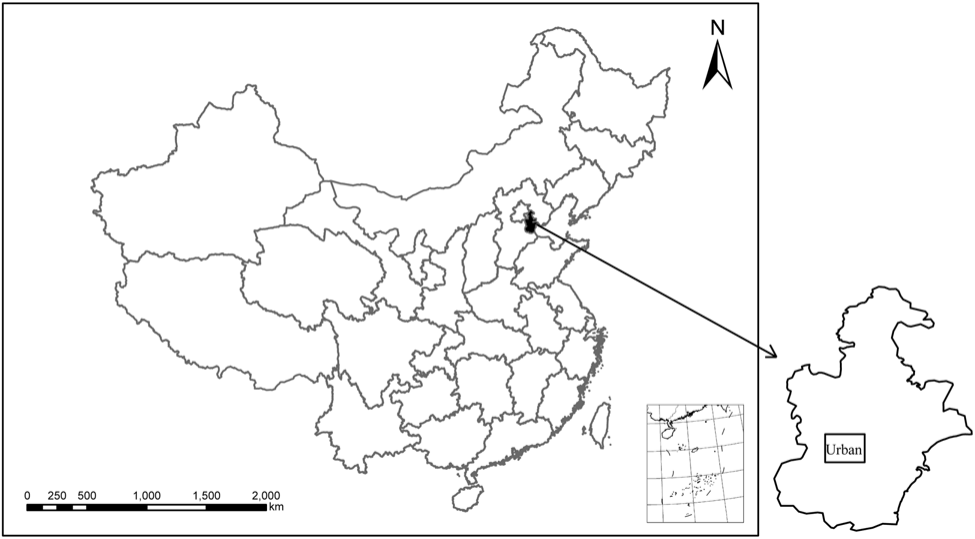
The location of study area in China and the site of selected buildings in Tianjin City.

Tianjin is the second largest city by urban population after Beijing in Northern China (Fig 1, 39°10′ N, 117°10′E), with a population of approximately 10 million in 11, 919 km^2^. It is located in the north of China, on the lower reaches of the Haihe River and is adjacent to the Bohai Sea. Tianjin has a semi-moist continental monsoon climate, with monsoon prevailing all year round and distinct four seasons. The annual precipitation of Tianjin city is 570 mm, most of which occurs during June to August. The mean annual temperature is 12.3°C, with annual extremes of 41.7°C and -23.3°C.

### Selection of buildings

To reveal the effect of climate on energy consumption for different types of buildings, residential, commercial and large venue buildings in the central urban area of Tianjin (Fig 1) were selected. No specific permissions are required for this city and these selected building types because the selected study area does not belong to protected area or private land. Also, there are not endangered or protected species. The residential buildings generally uses coal for winter heating, whereas natural gas and electricity for both heating and cooling of commercial and large venue buildings, respectively. In the present study, a 9-storey residential building with a total area of 2, 790 m^2^ and a 2.8 m floor-to-floor height was selected for energy consumption simulation. A generic commercial building (year-round use with air-conditioning) was selected. It is a 5-storey building with a curtain walling design, a 5 m floor-to-floor height and a total gross floor area of 17, 024 m^2^. The selected large venue building has a 4.8 m floor-to-floor height and a total gross floor area of 25, 900 m^2^. A summary of the key design parameters is shown in Table 1.

**Table 1:** Design data for the selected commercial, large venue and residential buildings.

Building type	Building envelope HTC (w/m^2^°C)			Indoor design condition Summer/winter			Internal load density			Window-to-wall ratio			
	Wall	Roof	Floor	T	RH	ACR	Occupancy	Lighting	Equipment	East	South	West	North
				(°C)	(%)	(1/h)	(m^2^/person)	(W/m^2^)	(W/m^2^)				
Commercial	0.53	0.48	2.04	25/18	60/30	1.5/1.5	3–4	13	13	0.48	0.46	0.30	0.29
Large venue	0.57	0.48	2.04	26/20	60/30	0.36	8–16.5	15	17	0.30	0.32	0.30	0.28
Residential	0.55	0.46	0.49	18	30	0.51	12	1.3	2.5	0.18	0.48	0.18	0.25

Note: HTC, heat transfer coefficient; T, Temperature; RH, Relative humidity; ACR, Air change rate

### Multiyear simulation of building energy consumption

Energy consumption simulation was carried out using the TRNSYS software. The TRNSYS is transient system simulation program with a modular structure that was designed to solve complex energy system problems by breaking complex problem down into a serious of smaller components [21]. The smaller components will be integrated together as a visual interface known as the TRNSYS Simulation Studio, and building parameters are inputted through a dedicated visual interface. TRNSYS has been commercially available since 1975 and is widely used for simulating solar energy applications, conventional buildings, and even biological processes.

Besides building input data, weather data files was also built into the software as an input file. To obtain the hour-by-hour energy consumption during the 30 years (1981–2010), data for multiple climatic variables in the form of 8760 hourly records per variable (dry bulb temperature, DBT; wet bulb temperature, WBT; global solar radiation, GSR; wind speed, WS; and wind direction, WD) for each year from 1981 to 2010 rather than typical meteorological year (TMY) data were required for the energy simulation. The data of required climatic variables is from Tianjin Meteorological Information Center and the meteorological observation station is located in the urban center of city (Fig 1). The homogeneity of the selected meteorological data has been strictly tested to ensure the reliability and accuracy of data. The WS and WD were directly collected from observed data. The dry-bulb temperature and relative humidity recorded for 4 times per day were used to generate the hourly data through cubic spline interpolation and then to permit calculation of the wet-bulb temperature. The hourly solar radiation was first generated from daily total solar radiation by using the Collores-Perein and Rabl model and then adjusted for rainy, foggy and sunny weather conditions. Comparisons of the generated hourly dry-bulb temperature, relative humidity and solar radiation with the observed hourly data from 5 recent years (2006–2010) were conducted to verify the reliability of the generated data. The building heating (in the winter period from 15 November to 15 March in the next year) and cooling (in the summer period from 1 June to 30 August) energy consumption was analyzed and compared for each type of buildings.

### Monitoring of building energy consumption

**Fig 2:**
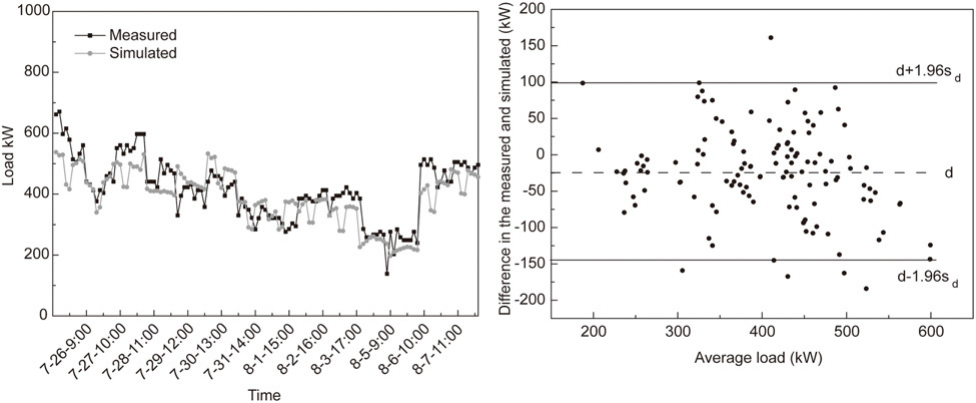
Comparisons between hourly measured and simulated cooling loads during the period from 26 July—7 August in 2010 (a) and the Bland-Altman plot of the measured and simulated loads (b). The upper and lower solid lines in Fig 2b represent the limits of agreement, and the middle horizontal dashed line shows the mean differences.

To guarantee reliability of the simulated data, energy consumption of the three types of buildings was monitored during the heating period in 2010–2011 and the cooling period in 2010. The cooling energy consumption of commercial building was taken as an example to show the comparison between the measured and simulated energy consumption. Fig 2 showed that the measured and simulated energy consumption had very similar hourly pattern and very small difference. Bland-Altman analyses revealed the good agreement between the measured and simulated energy consumption because the differences between the measured and simulated data is in a small range and only few points are outside the 95% limits of the agreement. This indicates the energy consumption simulation could be efficiently reflected the real energy consumption. Similar comparison results were found for other buildings.

### Determinant of extreme energy consumption

Percentile methods were used to determine the extreme energy consumption for building heating or cooling. Based on the number of energy consumption data, the 95^th^ percentile of daily energy consumption was defined daily extreme energy consumption and the 99^th^ percentile of hourly energy consumption was used to define the hourly extreme energy consumption.

### Statistical analysis

Simple linear regression analysis was performed to examine days of extreme energy consumption for the different kinds of buildings. As the climate parameters themselves and their interaction are critical for determining the impact on energy consumption, stepwise multiple linear regressions were used. Briefly, stepwise multiple linear regressions on the daily extreme energy consumption was performed against the possible climatic parameters, i.e., mean temperature, maximum temperature, minimum temperature, wet bulb temperature, solar radiation, sunshine duration, and wind speed. Correlation analysis was used to examine the effects of climatic parameters on hourly extreme energy consumption and further determine if there were delays in the effects of climatic parameters on hourly extreme energy consumption. Statistical analyses were performed using SPSS 11.0 for Windows, and significance levels were set at *P* < 0.05.

## Results

### Variations of days of extreme energy consumption for different types of buildings

**Fig 3:**
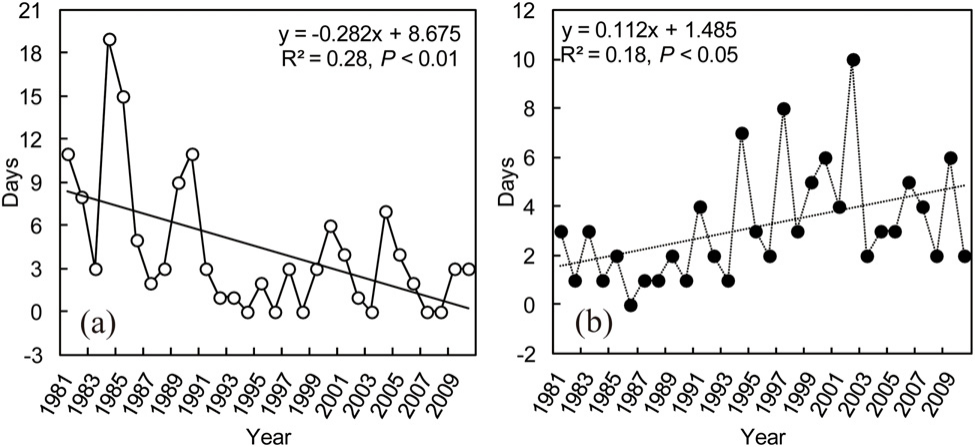
Yearly variations in days for extreme heating (a) and cooling (b) energy consumption of large venue building.

Days of extreme heating energy (electricity) consumption of large venue building showed large and significant decrease from 1981 to 2010 (R^2^ = 0.28, *P* < 0.01) (Fig 3a), with a decreasing rate of average 2.8 d/10a. By contrast, the days of extreme cooling energy (electricity) consumption had significant increase during the recent 30 years (R^2^ = 0.18, *P* < 0.05) (Fig 3b). The increasing rate (1.1 d/10a) of extreme cooling energy consumption is lower than the decreasing rate of extreme heating energy consumption. In addition, large interannual fluctuations were found for the days of extreme heating or cooling energy consumption, with the highest days of extreme heating (19d) and cooling (10d) energy consumption occurring in 1984 and 2002, respectively.

**Fig 4:**
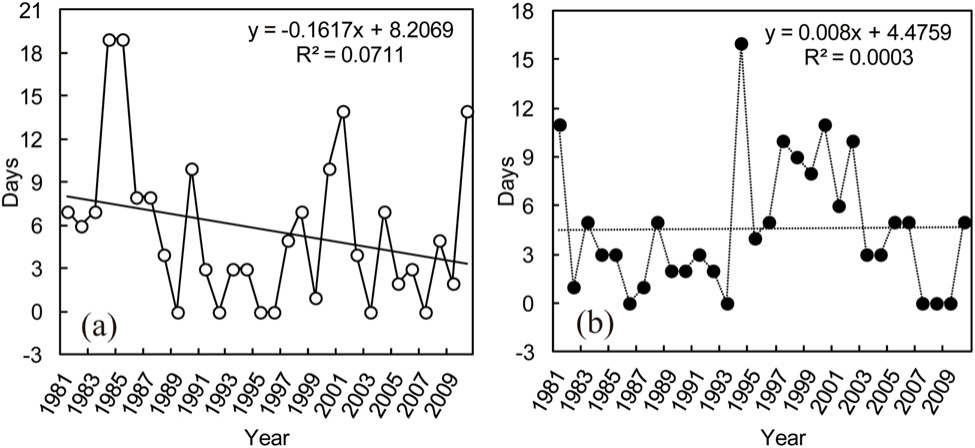
Yearly variations in days for extreme heating (a) and cooling (b) energy consumption of commercial building.

**Fig 5:**
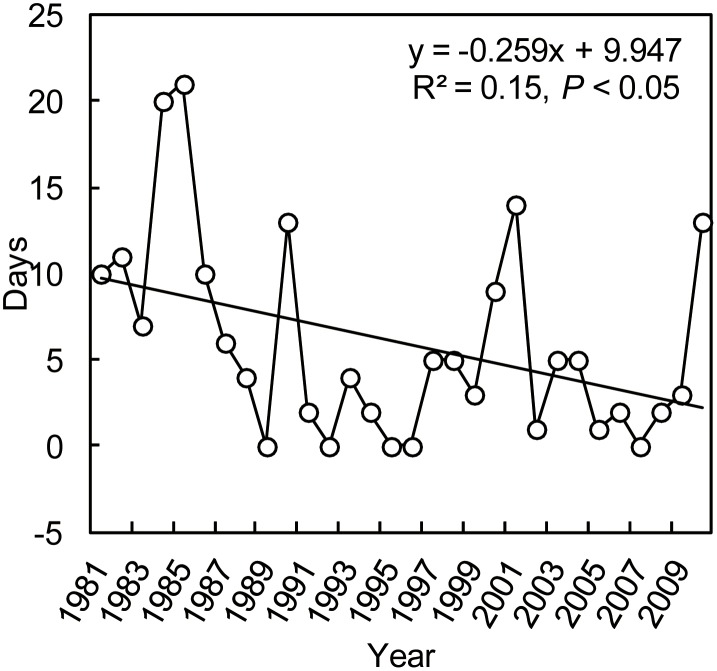
Yearly variations in days for extreme heating (a) and cooling (b) energy consumption of residential building.

For commercial building, days of extreme heating or cooling energy (natural gas) consumption did not have significant variations from 1981 to 2010 (*P* > 0.05) (Fig 4) although there was a weak decrease (1.6 d/10a) in the extreme heating energy consumption (Fig 4a). There were large interannual fluctuations for the days of extreme heating and cooling energy consumption. The highest days of extreme heating (19d) energy consumption occurred in 1984 and 1985, and extreme cooling (16d) energy consumption was in 1994. The same to large venue building, days of extreme heating energy (coal) consumption of residential building showed significant decrease during the last 30 years (R^2^ = 0.15, *P* < 0.05) (Fig 5), with the decreasing rate of 2.59 d/10a. There were also interannual fluctuations for the days of extreme heating energy consumption, with the highest days of extreme heating (21d) in 1985.

### Climate impacts on daily extreme energy consumption

Table 2 showed the correlations between daily extreme energy consumption and climatic parameters for the selected different types of buildings. Daily extreme heating or cooling energy consumptions for large venue building were not impacted by climate parameters except a weakly negative correlation between extreme heating energy consumption and minimum temperature (Table 2). By contrast, daily extreme heating energy consumption for commercial or residential buildings and daily extreme cooling energy consumption for commercial building had significant correlations with various climatic parameters (Table 2).

**Table 2:** Correlations between daily extreme energy consumption and climatic parameters for different kinds of buildings.

Building types		DBT	WBT	SR	WS	MIT	MAT
Commercial	Heating	-0.576**	-0.416**	0.021	0.044	-0.357**	-0.579**
	Cooling	0.439**	0.622**	0.034	0.055	0.381**	0.342**
Large venue	Heating	-0.172	-0.104	0.033	-0.094	-0.202*	-0.091
	Cooling	-0.077	-0.126	0.078	-0.075	-0.103	-0.087
Residential	Heating	-0.908**	-0.785**	0.156*	0.041	-0.715**	-0.634**

Note:

* *P* < 0.05;

** *P* < 0.01;

DBT, dry bulb temperature; WBT, wet bulb temperature; SR, solar radiation; WS, wind speed; MIT, minimum temperature; MAT, maximum temperature. The same below.

In order to reveal the dominant climatic parameters impacting daily extreme energy consumption, stepwise multiple regressions were performed. For commercial building, MAT, DBT, SR and MIT entered the regression model for the analysis of daily extreme heating energy consumption (Table 3). In addition, with the increase of climatic parameters, the R^2^ showed apparent increase from 0.33 to 0.71, indicating these climatic parameters together can explain 71% of the variations of daily extreme heating energy consumption. Daily extreme cooling energy consumption for commercial building was only affected by the WBT that could explain 38% of the variations of daily extreme cooling energy consumption (R^2^ = 0.382) (Table 3).

**Table 3:** Regression analysis for daily extreme energy consumption of commercial building against the climatic parameters.

	One-factor model	Two-factor model	Three-factor model	Four-factor model
Heating	-27.68×MAT	-17.03×MAT	-9.86×MAT	-1.95×MAT
		-16.78×DBT	-48.69×DBT	-75.17×DBT
			-20.79×SR	-20.58×SR
				16.844×MIT
Constant	931.09	853.37	794.98	803.27
R^2^	0.332***	0.399***	0.677***	0.715***
Cooling	51.48×WBT			
Constant	-69.15			
R^2^	0.382***			

Note:

*** *P* < 0.001

Regression analysis suggests the daily extreme heating or cooling energy consumptions for large venue building is not related to climatic parameters although there was a significant relationship between the daily extreme value and MIT at the 0.05 significant level (Table 4, R^2^ = 0.033). Four climatic parameters (DBT, MIT, MAT, WS) entered the regression model for the daily extreme heating energy consumption of residential building, but the R^2^ did not show apparent increase from one-factor model to four-factor model (Table 5). This indicates the daily extreme heating energy consumption for residential building is dominantly affected DBT and DBT can explain 82% of the variations of extreme value (R^2^ = 0.824).

**Table 4:** Regression analysis for daily extreme energy consumption of large venue building against the climatic parameters.

	One-factor model
Heating	-2.78×MIT
Constant	3367.84
R^2^	0.033*
Cooling	NS

Note:

* *P* < 0.05;

NS, no significance

**Table 5:** Regression analysis for daily extreme energy consumption of residential building against the climatic parameters.

	One-factor model	Two-factor model	Three-factor model	Four-factor model
Heating	-13.05×DBT	-11.02×DBT	-6.49×DBT	-7.57×DBT
		-2.37×MIT	-4.36×MIT	-3.71×MIT
			-2.50×MAT	-2.31×MAT
				-1.34×WS
Constant	242.33	231.40	235.13	238.15
R^2^	0.824***	0.850***	0.866***	0.874***

Note:

***P < 0.001

### Climatic impacts on hourly extreme energy consumption

The correlation analysis between hourly extreme energy consumption and climatic parameters were shown in Table 6. Hourly extreme heating energy consumption had negative correlations with DBT, WBT and WS in all the three types of buildings. The correlation coefficient with DBT was higher than WBT. This indicates hourly extreme heating energy consumption is dominantly affected by the DBT, followed by WBT. The WS has little effect due to the very small correlation coefficient. The hourly extreme cooling energy consumption for commercial building had positive correlations with DBT, WBT and SR, with the highest correlation coefficient with WBT, indicating the hourly extreme cooling energy consumption is mainly impacted by WBT. For large venue building, hourly extreme cooling energy consumption may not be affected by climatic parameters because the correlation coefficient was very small although significant correlations were found.

**Table 6:** Correlations between hourly extreme energy consumption for different types of buildings and climatic parameters.

Building types		time	DBT	WBT	SR	WS
Commercial	Heating	On time	-0.415**	-0.288**	0.089**	-0.037
		1 hour before	-0.444**	-0.314**	0.018	-0.056*
		2 hour before	-0.437**	-0.324**	-0.089*	-0.077**
		3 hour before	-0.407**	-0.317**	-0.172**	-0.087**
	Cooling	On time	0.292**	0.613**	0.111**	-0.010
		1 hour before	0.223**	0.542**	0.090**	-0.003
		2 hour before	0.149**	0.436**	0.045	-0.019
		3 hour before	0.095**	0.338**	-0.023	-0.019
Large venue	Heating	On time	-0.338**	-0.307**	0.082**	-0.078**
		1 hour before	-0.352**	-0.318**	-0.043	-0.091**
		2 hour before	-0.351**	-0.315**	-0.141**	-0.098**
		3 hour before	-0.341**	-0.303**	-0.204**	-0.098**
	Cooling	On time	0.011	-0.090**	-0.131**	-0.037
		1 hour before	0.028	-0.101**	-0.048	-0.041
		2 hour before	0.031	-0.106**	0.044	-0.019
		3 hour before	0.030	-0.093**	0.104**	-0.037
Residential	Heating	On time	-0.715**	-0.694**	0.048	-0.032*
		1 hour before	-0.871**	-0.805**	0.006	-0.038*
		2 hour before	-0.902**	-0.820**	-0.029	-0.040**
		3 hour before	-0.831**	-0.767**	-0.059**	-0.032*

Note:

* P < 0.05;

** *P* < 0.01

For commercial building, the correlation coefficient between hourly extreme heating energy consumption and DBT and WBT showed weak increase from the climatic parameters on time to the climatic parameters 1 or 2 hour before, indicating that the climate impact on the hourly extreme heating energy consumption has about 1 or 2 hour delay. The correlation analysis for large venue building suggests that there are 1–2 hour delay for the DBT and WBT impacts and 3 hour delay for the SR impact. The DBT and WBT impacts on hourly extreme heating energy consumption for residential building have 2 hour delay. Although there were significant impacts of WBT and DBT from on time to 3 hour before on the hourly extreme cooling energy consumption, the correlation coefficient weakly decreased, indicating no apparent delay for the climate impacts on the hourly extreme cooling energy consumption.

## Discussion and Conclusions

The paper simulated heating and cooling energy consumption for three types of buildings by TRNSYS software and the simulated data were compared with measured data to guarantee reliability of the simulated data. The 95^th^ and 99^th^ percentile of obtained energy consumption were used to define the daily and hourly extreme energy consumption, respectively. The climate impacts on daily and hourly extreme heating and cooling energy consumption were determined.

Days of extreme heating energy consumption generally showed decreasing trend in the last 30 years for all the three types of buildings although the decreasing trend is not significant at the 0.05 level for the commercial building. The decreasing trend is consistent with the variations of average heating energy consumption in previous studies that determined the energy consumption by using degree-days [4,6,19]. Tianjin, especially in the urban area, has experienced substantial increase in temperature in the last decades due to the rapid urbanization [16,20]. With the continuous increase of greenhouse and urban expansion, the climate in the large city will undoubtedly show apparent warming in the future, as pointed by Argüeso *et al*. (2014) who concluded the temperature increase by the combined effects of GHGs and urban expansion could be double the increase due to the global warming alone at 2050 [22]. The continuously significant warming in urban climate will decrease the energy demand for building heating. These may be used to make measures for saving energy consumption of building heating at the conditions of higher indoor thermal comfort. In addition, the decrease of days of extreme heating energy consumption is beneficial for safety operation of Heating systems because the occurrence of extreme energy consumption may be further less in the future. However, it is necessary to note that although the days of extreme heating energy consumption decreased, there are large interannual fluctuations that should be taken account to design the capacity of Heating systems.

The days of extreme cooling energy consumption, difference from heating energy consumption, showed very significant increase (large venue building) or no variation but with very large interannual fluctuations (commercial building). Just pointed out above, significant warming will continue in northern China in the future [23], especially in the urban areas of large cities due to the increase of greenhouse and urban expansion [22]. This will lead to significant increase or large interannual fluctuations of extreme cooling energy consumption as well as increasing average energy consumption, which should fully be considered when correcting or optimizing Air conditioning system design because the increase in energy consumption, especially extreme energy consumption may have risen to values above design conditions of Air-conditioning systems of buildings.

From the correlation and regression analyses, it is found that the relationships of daily extreme energy consumption to climate parameters vary with the types of buildings. Daily extreme heating or cooling energy consumption for large venue building is not affected by climate parameters. Daily extreme heating energy consumption for commercial buildings is closely related with MAT, DBT, SR, MIT, whereas it is only impacted by DBT for residential building. For the extreme cooling energy consumption of commercial building, the WBT is the dominant affecting climatic parameter, which means that the cooling energy consumption is impacted by the combination of temperature and humidity. The hourly extreme heating energy consumption is dominantly impacted by DBT, followed by WBT for all the three types of buildings, whereas cooling energy consumption is closely related with WBT for commercial building or has no apparent relationship to climate for the large venue building. The climate impacts on hourly extreme heating energy consumption have 1–3 hour delay for three buildings, dependent of climatic parameters. This information may be useful for giving a basis for energy-saving or adjusting operation of Heating or Air conditioning systems when taking into account the climate impacts.

In summary, the climate impacts on extreme energy consumption of different types of buildings are determined in this study. Days of extreme heating energy consumption generally showed decreasing trend in the last 30 years and days of extreme cooling energy consumption showed increase or no variation. However, large interannual fluctuations exist in the extreme heating and cooling energy consumption. This would be helpful for researchers or designers to make measures for improving energy efficiency or ensuring the safety of Heating or Air conditioning systems. The impacts of climate on the extreme energy consumption depend on the building types or climatic parameters. This would be of interests to policy makers and building industry managers on how to make appropriate measures improve operating efficiency of Heating or Air conditioning systems and ensure their operating safety at the conditions of changing climate.

## Supporting Information

S1 File Raw data. Measured and simulated energy consumption data.
(XLS)

S2 File Raw data. Days for extreme energy consumption of three types of buildings.
(XLS)

S3 File Raw data. Daily extreme energy consumption of different types of buildings.
(XLS)

S4 File Raw data. Hourly extreme energy consumption of different types of buildings.
(XLS)
